# Protein-protein interfaces are vdW dominant with selective H-bonds and (or) electrostatics towards broad functional specificity

**DOI:** 10.6026/97320630013164

**Published:** 2017-06-30

**Authors:** Christina Nilofer, Anshul Sukhwal, Arumugam Mohanapriya, Pandjassarame Kangueane

**Affiliations:** 1 Biomedical Informatics (P) Ltd, Irulan Sandy Annex, Puducherry 607 402, India; 2 National Centre for Biological Sciences, TIFR, UASGKVK Campus, Bangalore, Karnataka, India; 3 School of Bio Sciences and Technology, VIT University, Vellore, Tamil Nadu, India; 4 SASTRA University, Thanjavur, Tamil Nadu, India

**Keywords:** PPI, interface, energy, molecular function, van der Waals (vdW), hydrogen bonds (H-bonds), electrostatics

## Abstract

Several catalysis, cellular regulation, immune function, cell wall assembly, transport, signaling and inhibition occur through Protein- Protein Interactions (PPI). This is possible with the formation of specific yet stable protein-protein interfaces. Therefore, it is of interest to understand its molecular principles using structural data in relation to known function. Several interface features have been documented using known X-ray structures of protein complexes since 1975. This has improved our understanding of the interface using structural features such as interface area, binding energy, hydrophobicity, relative hydrophobicity, salt bridges and hydrogen bonds. The strength of binding between two proteins is dependent on interface size (number of residues at the interface) and thus its corresponding interface area. It is known that large interfaces have high binding energy (sum of (van der Waals) vdW, H-bonds, electrostatics). However, the selective role played by each of these energy components and more especially that of vdW is not explicitly known. Therefore, it is important to document their individual role in known protein-protein structural complexes. It is of interest to relate interface size with vdW, H-bonds and electrostatic interactions at the interfaces of protein structural complexes with known function using statistical and multiple linear regression analysis methods to identify the prominent force. We used the manually curated non-redundant dataset of 278 hetero-dimeric protein structural complexes grouped using known functions by Sowmya et al. (2015) to gain additional insight to this phenomenon using a robust inter-atomic non-covalent interaction analyzing tool PPCheck (Anshul and Sowdhamini, 2015). This dataset consists of obligatory (enzymes, regulator, biological assembly), immune and nonobligatory (enzyme and regulator inhibitors) complexes. Results show that the total binding energy is more for large interfaces. However, this is not true for its individual energy factors. Analysis shows that vdW energies contribute to about 75% ± 11% on average among all complexes and it also increases with interface size (r2 ranging from 0.67 to 0.89 with p<0.01) at 95% confidence limit irrespective of molecular function. Thus, vdW is both dominant and proportional at the interface independent of molecular function. Nevertheless, H bond energy contributes to 15% ± 6.5% on average in these complexes. It also moderately increases with interface size (r2 ranging from 0.43 to 0.61 with p<0.01) only among obligatory and immune complexes. Moreover, there is about 11.3% ± 8.7% contribution by electrostatic energy. It increases with interface size specifically among non-obligatory regulator-inhibitors (r2 = 0.44). It is implied that both H-bonds and electrostatics are neither dominant nor proportional at the interface. Nonetheless, their presence cannot be ignored in binding. Therefore, H-bonds and (or) electrostatic energy having specific role for improved stability in complexes is implied. Thus, vdW is common at the interface stabilized further with selective H-bonds and (or) electrostatic interactions at an atomic level in almost all complexes. Comparison of this observation with residue level analysis of the interface is compelling. The role by H-bonds (14.83% ± 6.5% and r2 = 0.61 with p<0.01) among obligatory and electrostatic energy (8.8% ± 4.77% and r2 = 0.63 with p <0.01) among non-obligatory complexes within interfaces (class A) having more non-polar residues than surface is influencing our inference. However, interfaces (class B) having less non-polar residues than surface show 1.5 fold more electrostatic energy on average. The interpretation of the interface using inter-atomic (vdW, H-bonds, electrostatic) interactions combined with inter-residue predominance (class A and class B) in relation to known function is the key to reveal its molecular principles with new challenges.

## Background

Protein complexes play an important role in catalysis, regulation, immunity, protein assembly, transport and inhibition through protein-protein interaction (PPI). This is fundamental to demonstrate a well-designed communicating network in biological systems. Interfaces are relevant in the context of targets defined for several diseases. The HIV-1 ENV GP160 (GP120/GP41) trimer spike [1], cholera toxin [2], α-integrin uPAR [3] and superoxide dismutase (SOD) [4] are some highlighted examples. These often include multiple protein subunits stabilized by several interfaces. Interface analysis is also contextual to fine tune interactions using holistic models involving networks data in the annotations of functional genomics initiatives [5]. Thus, the driving force deterministic of their interface features is essential for its molecular function. A number of features have been described since 1975 using simple dimer (two subunits) complexes. Our understanding of the interface has improved since then with increasing divergence and limited convergence. Interface residues are hydrophobic [6] and closely packed [7]. Hydrophobic residues are abundant in the interface than surface but less than the core [8]. Subsequently the use of hydrophobic mean-field potential in protein subunit docking was formulated [9]. In addition to hydrophobic patches in the interface [10], hydrogen bond and salt bridges [11,12,13] also stabilize the interface. Interfaces are made of aromatic and positively charged residues in certain complexes [14]. The conformational changes in the interface influence binding [15]. Residue propensity scores [16] and peptide segments [17] differentiated specific and non-specific complexes. Clusters of recognition sites [18] and conserved residues [19] at the interface are insightful. The difference in conserved residues at interface, core and surface is challenging [20]. Interfaces with less non-polar residues compared to surface [21,22] in addition to interfaces with more non-polar residues than surface are intriguing [8]. Description of interface area, hydrogen bonds, solvation free energy gain and binding energy to distinguish functional classes is impressive [23]. These observations have largely improved our understanding of the interfaces using 3 interfaces [6] in 1975 to 278 interfaces [23] in 2015. Conclusions drawn thus far are dependent on dataset size (number of complexes), type (homo, hetero, mixed) and analysis methods (residue or atomic models). However, there is further scope for the improved understanding of this phenomenon. The stability of interface is usually dependent on the proportion of residues (thereafter referred as interface size) buried between subunits [24] and its corresponding interface area [6, 8]. Nonetheless, the role played by vdW in relation to known molecular function is not explicitly analyzed and reported. Therefore, it is of interest to use a manually curated non-redundant dataset of 278 heterodimer subunit interfaces as described elsewhere [23] to relate interface size with vdW, H-bond and electrostatic energy to gain further insights using PPCheck (a robust tool for inter-atomic interface analysis) [25, 26]. This analysis is restricted to hetero complexes for the purpose of clarity and comparison with previously known information. It should be noted that homo (identical subunits) dimer complexes stabilized by interfaces with unique characteristics in a completely different platform as described elsewhere [27] is not included in this study.

## Methodology

### Dataset

**Figure 1:**
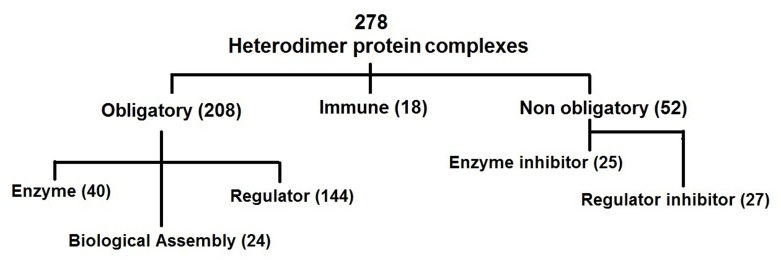
Grouping of a non-redundant dataset of 278 heterodimer protein complexes into functional groups as described elsewhere [23]. These include obligatory (208), immune (18) and non-obligatory (52). The obligatory protein complexes are further classified into enzyme (40), regulator (144) and biological assembly (24) and the non-obligatory protein complexes into enzyme inhibitor (25) and regulator inhibitor (27).

We used a dataset of 278 protein complexes as described elsewhere by Sowmya et al. (2015) [23]. It consists of 40 Enzymes, 144 Regulatory, 25 Enzyme inhibitors, 27 Regulatory inhibitors, 18 Immune complexes and 24 biological assembly complexes (Figure 1). This dataset is similar to a manually curated dataset having functional annotations described earlier by Sowmya et al. (2011) [21]. We further grouped complexes associated with regulator, enzyme and biological assemblies as obligatory (essential) and those of enzyme and regulatory inhibitors as nonobligatory (unwanted). Thus, there are 208 obligatory, 52 nonobligatory and 18 Immune complexes in the dataset Figure 1. The structure data for protein-protein complexes is made available for public download at http://bioinformation.net/ppi/

### PPCheck, an interface analysis tool

PPCheck (Anshul and Sowdhamini, 2013; Anshul and Sowdhamini, 2015) (freely available at http://caps.ncbs.res.in/ ppcheck/) [25, 26] is a server, which identifies non-covalent interactions based on distance between atoms of the two interacting proteins. In PPCheck, two residues between binding proteins are considered to be interacting if the distance between their atom(s) is less than the cut-off distance. This cut-off distance [26] varies for various non-covalent interactions (hydrogen bonds, electrostatic and vdW) as implemented in PPCheck [25, 26]. These interactions are subsequently converted into pseudoenergies using various force fields as described elsewhere [25]. It should be noted that the role of water is neglected in the analysis.

### Interface and Energy Analysis

Interface size (number of interface residues) and energies associated with various interactions (vdW, H-bonds, electrostatic) were calculated using PPCheck for each of 278 complexes. This data is presented as supplementary material 97320630013164S1 in Microsoft office excel file format. The mean value for different energy components across different groups is given in Table 3. The list of r2 values among different groups is also given in Table 1 and Table 2.

### Caveat

It should be noted that electrostatic energy was positive or unfavorable in few entries (Figure 3 and 4). This is due to strong force of repulsion between similarly charged residues (than the force of attraction between oppositely charged residues) at the interface or the number of similarly charged residues (and hence unfavorable interactions) was greater than oppositely charged residues at the interface. Hence, these effects were neglected in the analysis.

### Multiple Linear Regression Analysis

We performed multiple linear regression analysis of interface size with vdW, H-bonds, electrostatics and its total interface energies using Microsoft® Office Excel (version 2003) statistical analysis tool (regression). Its co-efficient of determination (r2), a predictive power score, was estimated with assessment of significance (pvalue) using statistical ANOVA test at 95% confidence limit.

## Results and Discussion

**Figure 2:**
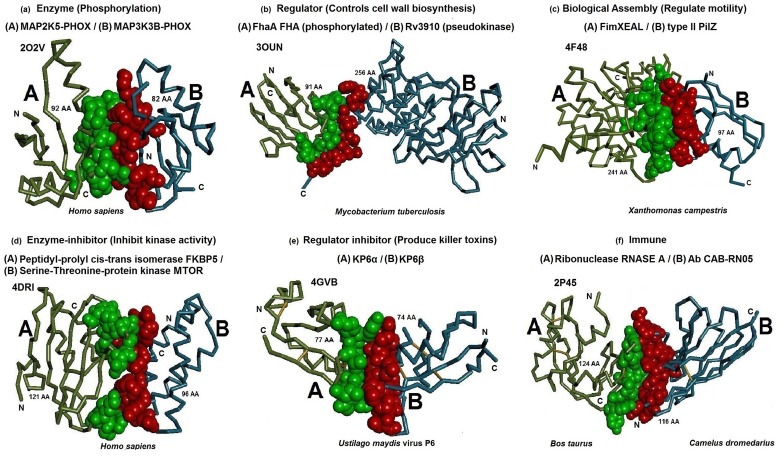
Protein-protein complexes among different categories are shown using Discovery studio™ [28] with backbone structures displayed in Ca stick style and interface regions depicted using CPK (Corey Pauling Koltun) representation. The interface residues of chain A and B are colorized in green and red, respectively. (A) An enzyme complex (PDB ID 2O2V) between MAP2K5-PHOX / MAP3K3B-PHOX, (B) A regulator complex (PDB ID 3OUN) between FhaA FHA protein/Rv3910, (C) A protein assembly complex (PDB ID 4F48) formed between FimXEAL / type II PilZ, (D) An enzyme-inhibitor complex (PDB ID 4DRI) between Peptidyl-prolyl cistrans isomerase FKBP5 / Serine-Threonine-protein kinase MTOR, (E) A regulator inhibitor complex (PDB ID 4GVB) between KP6α/ KP6β and (F) An immune complex (PDB ID 2P45) between Ribonuclease RNASE A / Ab CAB-RN05. The interface residues were identified using change in Accessible Surface Area (ASA) [29] upon complex formation as described elsewhere [8, 13].

PPI is an important phenomenon among several biological processes. It is associated with catalysis (e.g. phospho-rylation), regulation (e.g. controls cell wall biosynthesis), biological assembly (e.g. regulate motility), immune response (e.g. RNASE A / Ab CAB-RN05) and inhibition (enzyme inhibitor (e.g. inhibit kinase activity) and regulator inhibitor (e.g. produce killer toxins)) as shown in Figure 2. Therefore, the need to understand its molecular principles is imperative for engineering interfaces using site-directed mutagenesis for specific application. An understanding of its principles using known X-ray structural complexes is possible. This is often completed using inter-atomic [23] and inter-residue [6, 7, 8, 10, 11, 12, 21, 22] analysis of the interface. It is known that interfaces are hydrophobic in several complexes [6, 7, 8, 9]. However, H-bonds and salt bridges [10, 11, 13, 19] improve stability. The conformational stability of interfaces in HIV-1 ENV GP160 (GP120/GP41) trimer spike [1]; cholera toxin [2]; α- intergrin and uPAR [3] is a subject of debate over the last few decades towards the design of improved disease related targets. Hence, improved analysis, understanding, engineering, stability and functionality of the interface are largely enterprising in discovery platforms. We used a non-redundant manually curated dataset of 278 protein complexes to relate with different types of energies (vdW, H-Bonds, electrostatic) and interface size among solved structures with known function using PPcheck (a robust inter-atomic interface analysis tool). It should be noted that this dataset is unique with manually curated functional data grouped into categories as shown in Figure 1. This is a tedious and time consuming process. Moreover, the dataset consists of heterodimer complexes where the interacting subunits are non-identical (Figure 2). The complexes in the dataset where grouped based on their molecular function such as catalysis, regulator, biological assembly, immunity, enzyme inhibitors and regulator inhibitors (Figure 1). Further it is grouped into obligatory, non-obligatory and immune complexes. The dataset was also categorized into two independent classes based on residue level interface features as described elsewhere [21, 22] and as shown in Figure 6. Interface parameters such as interface size, binding energy, vdW energy, H-bond energy and electrostatic energies were compared using multiple regression analysis and its coefficient of determination (r2) was estimated among different functional groups and classes (Figure 7) of complexes.

**Figure 3:**
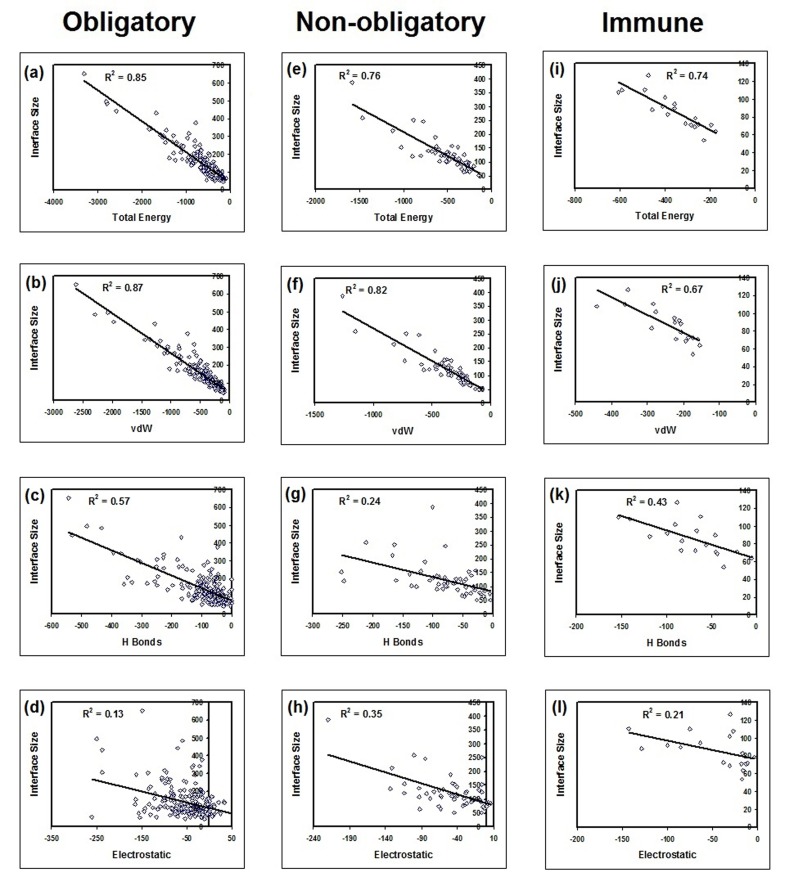
Correlation between interface size and energy (total, van der Waals, hydrogen bond and electrostatic) is shown. The correlation of determination r2 was calculated for energy and interface size among obligatory (compulsory), non-obligatory and immune complexes.

**Figure 4:**
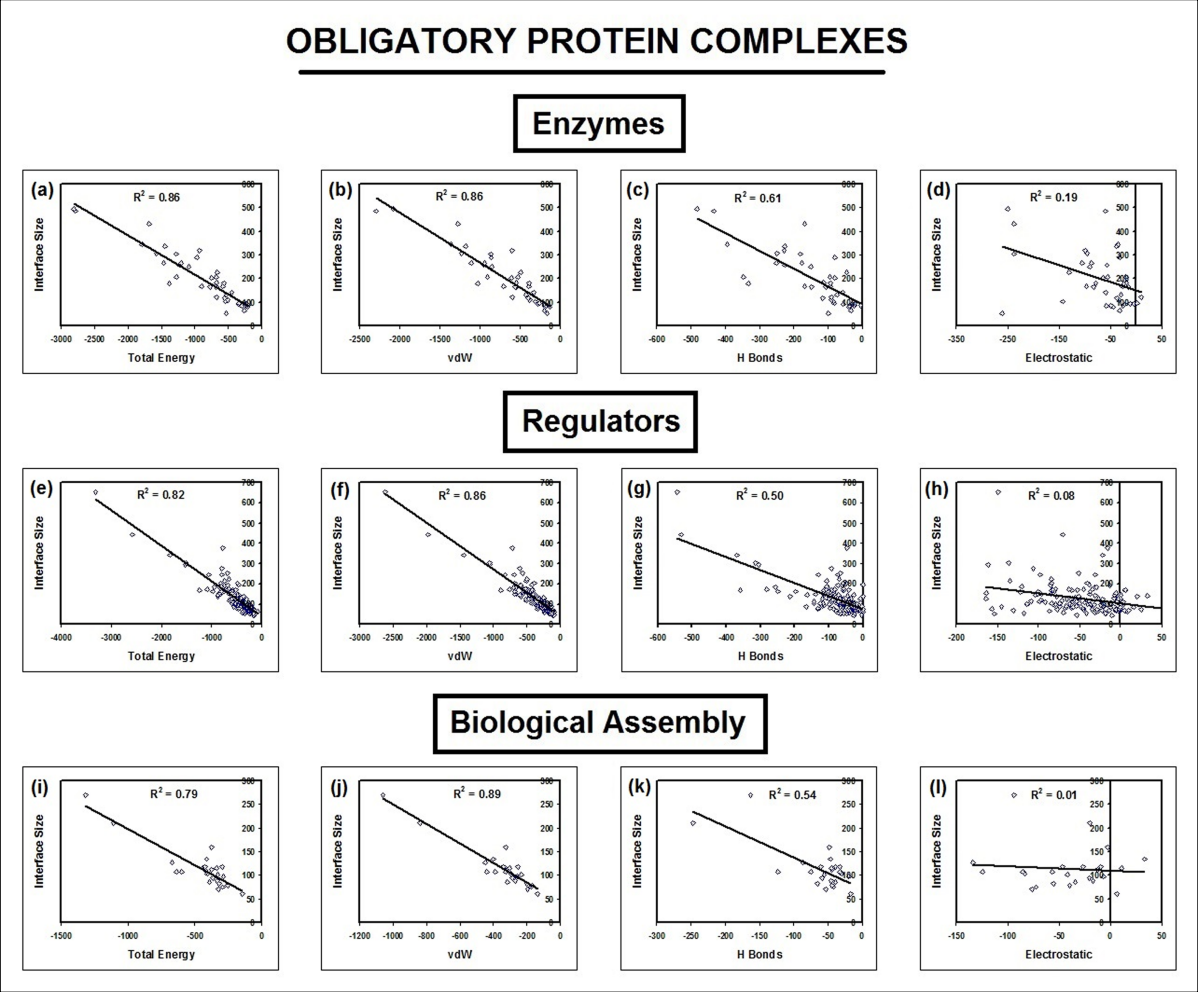
Correlation between interface size and energy (total, van der Waals, hydrogen bond and electrostatic) is shown. The correlation of determination r2 was calculated for energy and interface size among enzymes, regulators and biological assemblies.

**Figure 5:**
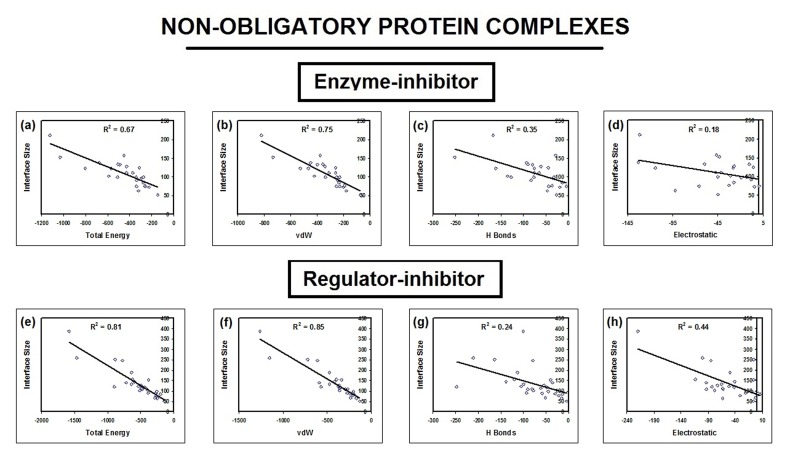
Correlation between interface size and energy (total, van der Waals, hydrogen bond and electrostatic) is shown. The correlation of determination r2 was calculated for energy and interface size among enzyme inhibitors and regulator inhibitors.

**Figure 6:**
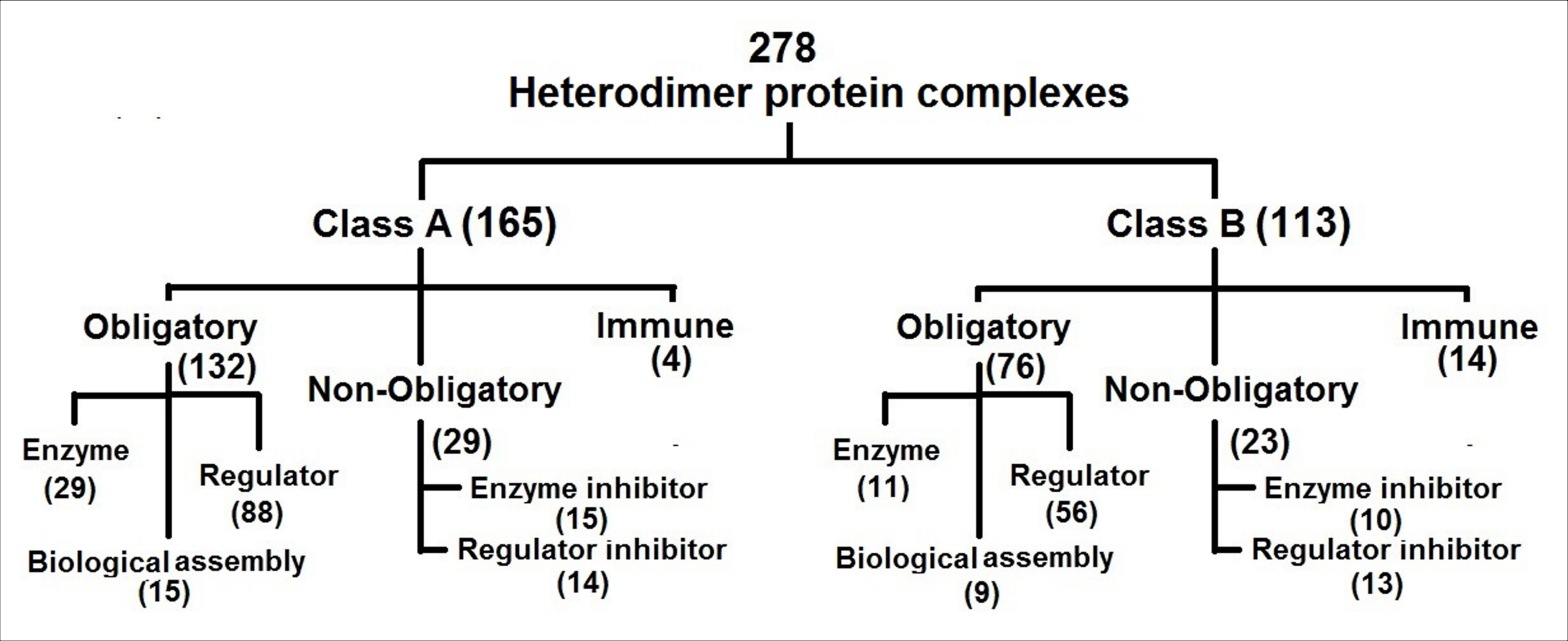
Grouping of 278 hetero-dimer non-redundant dataset protein complexes into class A (interface non-polar residues is more than surface) and class B (interface non-polar residues less than surface and core) [21, 22]. Class A (165) protein complexes are further grouped into functional groups such as obligatory (132), immune (4) and non-obligatory (29). The obligatory protein complexes are further classified into enzyme (29), regulator (88) and biological assembly (15) and the non-obligatory protein complexes into enzyme inhibitor (15) and regulator inhibitor (14). Simultaneously class B (113) is grouped into obligatory (76), immune (14) and non-obligatory (23). The obligatory protein complexes are further classified into enzyme (11), regulator (56) and biological assembly (9) and the nonobligatory protein complexes into enzyme inhibitor (10) and regulator inhibitor (13).

**Figure 7:**
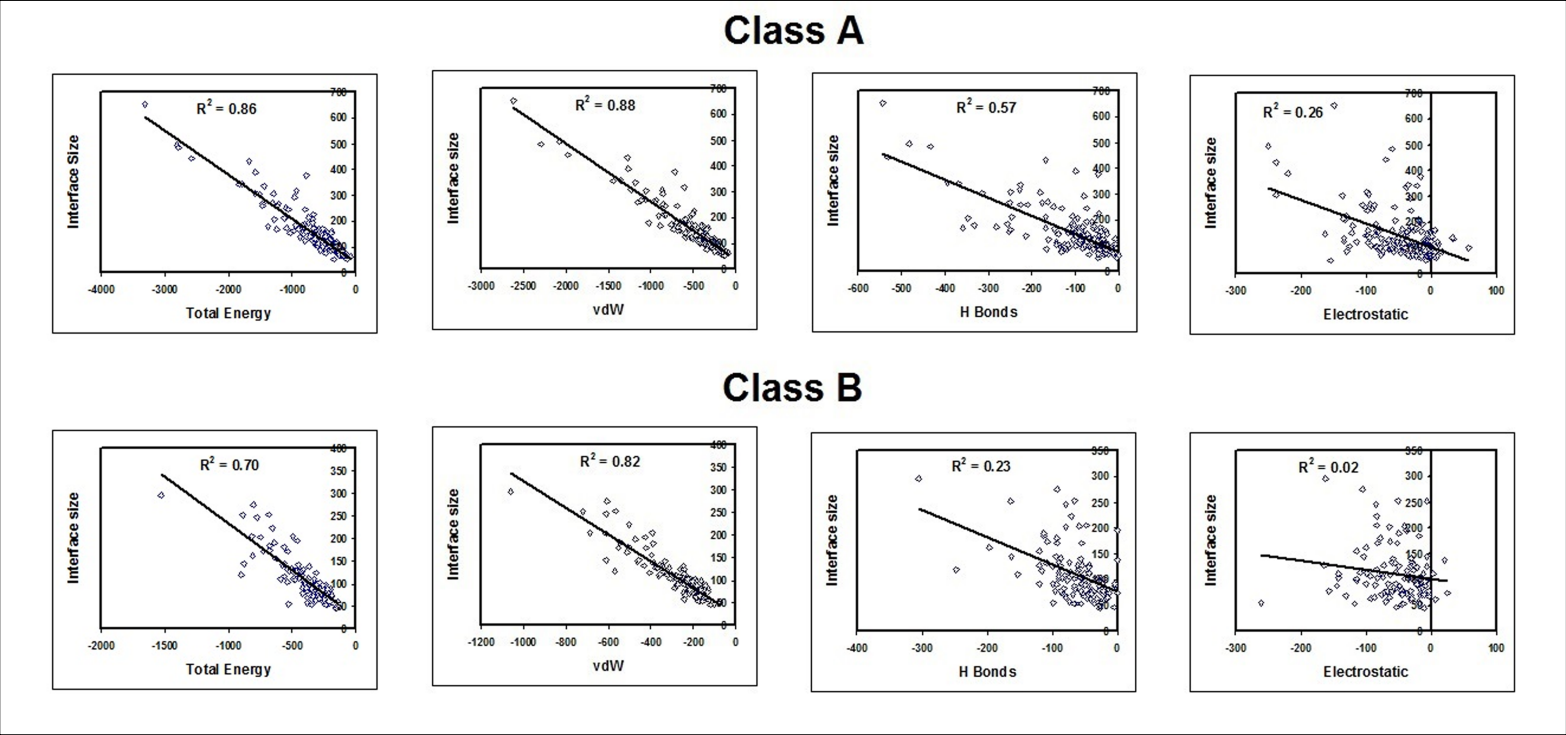
Correlation between interface size and energy (total, van der Waals, hydrogen bond and electrostatic) is shown. The correlation of determination r2 was calculated for energy and interface size among class A (interface non-polar residues is more than surface) and class B (interface non-polar residues less than surface) [21].

There are 278 interfaces in the dataset and it is non-redundant, comprehensive and representative. Each interface is different in its absolute view. However, there are common patterns or features among them. Gleaning their common features across different interfaces is the bottleneck. The binding of two proteins is related to interface size (number of interface residues involved in binding) [24] and its corresponding interface area [6, 8] related to total interface energy. This total energy is composed of vdW, H-bonds and electrostatic energy. The fractional (%) and its proportional distribution of each of these energies to interface size in each of these complexes are characteristics of the interface. Hence, it is of interest to relate interface size to energy (vdW, Hbonds, electrostatic) corresponding to several non-covalent interactions at the interface among different functional groups (Figure 1) and classes (Figure 6) of complexes. Previous analysis on this dataset reported the mean statistics of total energy, Hbonds and salt bridges [23]. However, this study did not explicitly document the role played by vdW in these interfaces. Our interest is to report the dominant and proportional effects of H-bonds, vdW and electrostatics using statistical and regression parameters. The co-efficient of determination (r2), a predictive power score with p-value using ANOVA test for each of the regression analysis is given in Table 1 and Table 2. Data in Table 1 in correspondence with Figures 3, 4, 5 and 7 shows that total and vdW energies increases with interface size independent of molecular function (Figures 3, 4 & 5) and interface residue preference (Figure 7) with p<0.01. Thus, vdW is the common factor with 75% ± 11% on average (Table 3) at the interface within atomic resolution. There is overlap between vdW and hydrophobic effects and this observation is in inferred concurrence as proposed elsewhere [6, 7, 8, 9]. However, the role by H - Bonds and electrostatic energy could not be ignored (Table 1 and 3). H-bonds increases with interface size among these complexes except for non-obligatory complexes (Table 1 and Figure 1c & Figure 1k) and (Figure 4 c, g, k) with an average influence of 15% ± 6.5%. Moreover, electrostatic energy increases with interface size among non-obligatory regulator inhibitors (Table 1 and Figure 5h) and its role is significant among this group as reported elsewhere [23]. It is interesting to note the percentage of electrostatic energy is almost non-existent on average in enzymes and enzyme inhibitors (Table 3). Table 4 shows examples of interesting interfaces where protein-protein binding occurs through vdW stabilized with H-bonds (28%) and without electrostatics (0%) in a protein transport complex (PDB ID: 3B0Z). There is also an example where the interface is largely vdW (92%) without H bonds (0%) and minimal electrostatics (8%) in a DNA binding protein complex (PDB ID: 3THO). Thus, the relationship between vdW and grouped molecular function is reported. Moreover, the specific role by either H-bonds and (or) electrostatics in most complexes is also described.

The importance of H-bonds (14.83% ± 6.5% and r2 = 0.61) among obligatory and electrostatic (8.8% ± 4.8% and r2 = 0.63) among non-obligatory within interfaces (class A) having more non-polar residues (Table 2) is adding value to the inference. This shows that H-bonds increases with size in obligatory complexes and electrostatics increases with size in non-obligatory complexes among non-polar interfaces (class A). This is not true among less non-polar interfaces (class B). However, interfaces (class B) with sub dominant non-polar residues show an average of 1.5 fold more electrostatic energy than the other class (class A except for immune) of complexes (Table 5). It should be noted that the preference for molecular function among residue level (classes A and B) complexes is unclear unlike atomic level interpretation where molecular function is related to H-bonds (obligatory and immune) and electrostatics (non-obligatory regulator-inhibitor). Thus, a combined observation of the interfaces in the context of known function using atomic and residue analysis provides additional insights towards the understanding of this phenomenon. Molecular functions are conserved in evolution and it is deterministic of structurally viable interfaces. The mechanism and hypothesis to describe gene fusion for conserved functions using evolved structural interfaces are known [30, 31]. The principles of PPI in the context of gene fusion leading to domain-domain interfaces are also compelling in this context.

## Conclusion

PPI is an important phenomenon in biological events such as catalysis, regulation, signaling, protein assembly, immune function and inhibition. Therefore, it is interest to understand its molecular principles using known structural complexes with defined molecular functions. The interface size (correspondingly interface area) is primarily deterministic of protein-protein binding. Inter-atomic level analyses show that vdW is the major contributor independent of molecular function. However, Hbonds are pronounced among obligatory and immune complexes unlike non-obligatory regulator inhibitor complexes with fitting electro-static energy. Thus, vdW is common at the interfaces with stabilizing H-bonds and electrostatic interactions with inferred specificity to molecular function. The corresponding strength of H-bonds and electrostatic interactions to interface size and its relation to grouped molecular function is of significance. The proportional presence of H-bonds in obligatory complexes and electrostatic in non-obligatory complexes among non-polar interfaces (class A) helps to integrate our interpretation to refine and design interfaces in the context of genetic variation, mutation and evolution in future investigations. The 150% increase in electrostatic energy among polar interfaces (class B) is providing better clarity to residue level analysis. It should be that PPcheck offers analysis of vdW, H-bonds and electrostatics energy in protein-protein interfaces. Hence, the overlap of vdW with Hbonds and electrostatics should be resolved in future. The degree of concurrence between vdW and hydrophobic effects should also be established for an integrated understanding of the phenomenon. We foresee more unambiguousness with additional structural data with known molecular function using improved analytical techniques.

## Critical comments

This manuscript analyzes the relationship between the size of protein interaction interfaces and several biophysical determinants of molecular interaction strength, namely van der waals forces, hydrogen bonds and electrostatics. To do this, the authors analyze a set of 278 interfaces published in 2015 by Sowmya et al. First, interfaces are annotated using an online tool called PPCheck, which returns interface size in amino acids, van der waals, hydrogen bond and electrostatic forces. Finally, the authors use regression to study the relationship between interface size and the different forces.

This work expands upon work published by Sowmya et al. by further investigating the relationship between interface size and components of binding energy, with van der waals and electrostatic forces not having been previously explicitly analyzed. In addition, the authors have further categorized the 278 interfaces as obligatory and non-obligatory, thereby allowing a new grouping of the proteins according to broader functional designation. The interface size calculated by PPCheck appears to be given in terms of number of amino acids rather than in terms of surface area as was used by Sowmya et al. (2015). Over all, the paper seems to reach many of the same conclusions as Sowmya et al. with new findings that van der waals forces correlate with interface size independent of molecular function, while hydrogen bonds and electrostatic forces appear to be correlated with interface size in different functional classes of proteins.

Overall, it is necessary to do something to further distinguish the current work from the previous work by Sowmya et al. The authors comment on the future knowledge to be gained from applying similar analyses to larger datasets. Based on the preliminary finding that the relationship between interface size and electrostatics has implications for protein function, why not download all co-crystal structures from the PDB, run them through PPCheck, then perform an unsupervised analysis to group complexes according to interface features, or the relationships between those features. Then the different groups can be surveyed to see which align to the functional groups that are currently being analyzed and the clustering can also be used to look for other common functional themes that might exist. This would potentially significantly extend the analysis without the need to carefully manually curate all of the complexes and group them prior to doing the analysis.

The authors provide p-values for the regressions, but do not directly compare two groups and test the difference in force contributions statistically. The authors observe that van der waals forces are not different between groups, but h-bonds are more pronounced in obligatory and immune complexes and electrostatic energy is more correlated with interface size in nonobligatory complexes. These differences should be evaluated statistically to show that they would not be expected under a null model that these interface size to force relationships are the same across groups. It is not clear whether the findings based on the 278 interfaces studied here will generalize to other complexes when grouped by similar functional category.

## Figures and Tables

**Table 1:** List of r2 values between interface size and energy among complexes of known function.

	Total Interface Energy		Van der Waals Energy		H Bond Energy		Electrostatic Energy	
	r2	p-value	r2	p-value	r2	p-value	r2	p-value
All complexes (278)	0.84	<0.01	0.87	<0.01	0.53	<0.01	0.15	<0.01
Obligatory (208)	0.85	<0.01	0.87	<0.01	0.57	<0.01	0.13	<0.01
Enzyme (40)	0.86	<0.01	0.86	<0.01	0.61	<0.01	0.19	0.01
Regulators (144)	0.82	<0.01	0.86	<0.01	0.5	<0.01	0.08	<0.01
Biological assembly (24)	0.79	<0.01	0.89	<0.01	0.54	<0.01	0	>0.01
Immune (18)	0.74	<0.01	0.67	<0.01	0.43	<0.01	0.21	>0.01
Non-obligatory (52)	0.76	<0.01	0.82	<0.01	0.24	<0.01	0.35	<0.01
Enzyme inhibitors (25)	0.67	<0.01	0.75	<0.01	0.35	<0.01	0.18	>0.01
Regulator inhibitors (27)	0.81	<0.01	0.85	<0.01	0.24	0.01	0.44	<0.01

**Table 2:** List of r2 values between interface size and energy among class A (interface non-polar residues more than surface) and class B (interface non-polar residues less than surface) [21, 22] protein complexes.

	Total Interface Energy		van der Waals Energy		H Bond Energy		Electrostatic Energy	
	r2	p-value	r2	p-value	r2	p-value	r2	p-value
Class A (165)	0.86	<0.01	0.88	<0.01	0.57	<0.01	0.26	<0.01
Obligatory (132)	0.87	<0.01	0.88	<0.01	0.61	<0.01	0.24	<0.01
Non-obligatory (29)	0.81	<0.01	0.86	<0.01	0.2	0.01	0.63	<0.01
Immune (4)	0.85	>0.01	0.72	>0.01	0.76	>0.01	0.02	>0.01
Class B (113)	0.7	<0.01	0.82	<0.01	0.23	<0.01	0.02	>0.01
Obligatory (76)	0.69	<0.01	0.81	<0.01	0.24	<0.01	0.02	>0.01
Non-obligatory (23)	0.7	<0.01	0.83	<0.01	0.3	<0.01	0.02	>0.01
Immune (14)	0.77	<0.01	0.77	<0.01	0.38	>0.01	0.28	>0.01

**Table 3:** Statistical analysis of different energies across different functional groups

	Electrostatic energy (%)		H Bond energy (%)		vdW energy (%)	
	Mean	SD	Mean	SD	Mean	SD
All complexes (278)	11.26	8.7	15.03	6.54	74.93	11.37
Obligatory (208)	11.48	9.09	14.72	6.55	75.28	11.75
Enzyme (40)	9.73	9.41	15.49	5.03	74.78	9.64
Regulators (144)	11.87	9.28	14.72	7.22	75.07	12.47
Biological assembly (24)	9.2	9.04	13.41	3.98	77.39	10.6
Immune (18)	12.16	8.82	19.07	6.46	68.77	10.78
Non-obligatory (52)	10.08	7.01	14.89	6.16	75.68	9.42
Enzyme inhibitors (25)	9.81	7.94	15.35	6.05	74.84	10.23
Regulator inhibitors (27)	9.94	6.29	14.47	6.34	76.45	8.73

**Table 4:** Examples of interesting interfaces

PDB ID	Function	Name	Electrostatic energy (%)	H-Bonds (%)	vdW energy (%)
3B0Z	Obligatory regulatory	Protein transport	0	27.72	72.28
3THO	Obligatory regulatory	DNA binding protein	8.28	0	91.72

**Table 5:** Statistical analysis of different energies across classes of complexes

	Mean	SD	Mean	SD	Mean	SD
Class A (165)	8.99	6.84	14.78	6.62	77.63	9.6
Obligatory (132)	8.83	7.06	14.83	6.5	77.84	9.41
Non obligatory (29)	8.76	4.77	14.14	6.81	78.12	8.77
Immune (4)	14.99	10.47	17.74	10.17	67.27	17.49
Class B (113)	14.33	9.96	15.4	6.42	70.99	12.58
Obligatory (76)	15.8	10.35	14.52	6.66	70.83	13.95
Non obligatory (23)	11.57	8.78	15.84	5.21	72.59	9.49
Immune (14)	11.35	8.56	19.45	5.48	69.2	8.97

## Supplementary Material

97320630013164S1
